# Redox Status and Proteostasis in Ageing and Disease

**DOI:** 10.1155/2016/7476241

**Published:** 2016-02-22

**Authors:** Federica Rizzi, Ioannis P. Trougakos, Gianfranco Pintus, Gerasimos P. Sykiotis

**Affiliations:** ^1^Department of Biomedical, Biotechnological and Translational Sciences, University of Parma, 43125 Parma, Italy; ^2^Department of Cell Biology and Biophysics, Faculty of Biology, National and Kapodistrian University of Athens, 15784 Athens, Greece; ^3^Laboratory of Cell Signaling and Redox Biology, Department of Biomedical Sciences, University of Sassari, 07100 Sassari, Italy; ^4^Service of Endocrinology, Diabetology and Metabolism, Lausanne University Hospital, 1011 Lausanne, Switzerland

An extensive network of components, generally referred to as the proteostasis network (PN), safeguards the functionality and integrity of the proteome, thus ensuring an optimal and efficient cell function. Failure of the PN represents a common trait of several chronic and age-related pathological conditions. This special issue features a collection of reviews and original articles covering distinct aspects of the effect of redox imbalance on the integrated complex of adaptive molecular signaling required to actively maintain proteome stability and functionality. The broad range of pathologies covered reflects appropriately the central relevance of the PN across different disease areas.

In their review entitled “It Is All About U(biquitin): Role of Altered Ubiquitin-Proteasome System and UCHL1 in Alzheimer Disease” A. Tramutola et al. discuss the impairment of the proteasome system as a consequence of oxidative stress and how this contributes to Alzheimer disease (AD) onset and progression. In the review entitled “Killing Me Softly: Connotations to Unfolded Protein Response and Oxidative Stress in Alzheimer's Disease,” B. Pająk et al. focused their attention on the possible causes of mitochondrial dysfunction in AD. Recent advances in the knowledge of mitochondria functions highlight that these organelles are extremely dynamic structures which not only represent the major bioenergetic hub of eukaryotic cells but also participate in the cellular signaling which control redox status and, likely, protein degradation. In “Cross Talk of Proteostasis and Mitostasis in Cellular Homeodynamics, Ageing, and Disease,” S. Gumeni and I. P. Trougakos review the functional cross talk of proteostasis (homeostasis of the proteome) and mitostasis (mitochondrial homeostasis) in the maintenance of cellular homeodynamics; they also refer to the impairment of mitochondrial quality control and how this impacts on proteome stability during ageing and/or age-related diseases. The biological significance of compounds that modulate the cellular redox state (i.e., oxidants and antioxidants), their roles in brain health, and the impact of redox modulation as well as potential uses and limitations of natural antioxidant compounds in selected neuropsychiatric disorders are discussed by E. A. Fraunberger et al. in their review entitled “Redox Modulations, Antioxidants, and Neuropsychiatric Disorders.” Oxidative stress has a major impact on the quality of membrane proteins highly expressed in the erythrocytes, which are required to preserve the structure and function of these cells. A. Pantaleo et al., in their original article entitled “Band 3 Erythrocyte Membrane Protein Acts as Redox Stress Sensor Leading to Its Phosphorylation by p^72^ Syk,” present experimental data to support the hypothesis that band 3 acts as redox sensor regulating its own phosphorylation and that substances leading to protracted phosphorylation of band 3 may trigger a cascade of events culminating in hemolysis. Advances in understanding myocardial redox signaling pathways and promising antioxidant therapeutic approaches that may beneficially impact on myocardial physiology are presented by A. Arcaro et al. in “Novel Perspectives in Redox Biology and Pathophysiology of Failing Myocytes: Modulation of the Intramyocardial Redox Milieu for Therapeutic Interventions—A Review Article from the Working Group of Cardiac Cell Biology, Italian Society of Cardiology.” The association between oxidative stress biomarkers and cardiovascular risk factors as well as left ventricular hypertrophy in children with chronic renal failure is demonstrated by D. Drożdż et al. in the original research article entitled “Oxidative Stress Biomarkers and Left Ventricular Hypertrophy in Children with Chronic Kidney Disease.” Finally, the paper from E. Al Jaaly et al. entitled “Pulmonary Protection Strategies in Cardiac Surgery: Are We Making Any Progress?” discusses the multifactorial mechanisms that relate to the activation of inflammatory and oxidative stress pathways and are involved in the development of pulmonary dysfunction.

The contributions selected for this special issue will concur to improve the understanding of the mechanisms that link failures of the cellular redox balance maintenance with the impairment of the protein quality control system, helping scientists to identify appropriate molecular targets for the development of new therapeutic strategies in the prevention and treatment of age-related diseases.

*Federica Rizzi* *Federica Rizzi* *Ioannis P. Trougakos* *Ioannis P. Trougakos* *Gianfranco Pintus* *Gianfranco Pintus* *Gerasimos P. Sykiotis* *Gerasimos P. Sykiotis*

